# Supplementary material for: Protective Effects of Astaxanthin on Ochratoxin A-Induced Liver Injury: Effects of Endoplasmic Reticulum Stress and Mitochondrial Fission–Fusion Balance
Source: Toxins (Basel). 2024 Jan 30;16(2):68. doi: 10.3390/toxins16020068 (PMC10893012; doi:10.3390/toxins16020068)
Supplement: Supplementary file 1 [file toxins-16-00068-s001.zip › toxins-2736813-SI.pdf]

Supplementary Materials: Protective Effects of Astaxanthin on Ochratoxin A-Induced Liver Injury: Effects of Endoplasmic Reticulum Stress and Mitochondrial Fission–Fusion Balance

Table S1. The primers that were applied in this research.

Primer	Sequence	Amplicon size (bp)
AHR-F	5′TTCAGGAAAGCAGAACAGCAA 3′	96bp
AHR-R	5′TCACAACTAATACGAAGCCAT 3′	
CAR-F	5′ACTTCACCTGCCCCTTTGCC 3′	105bp
CAR-R	5′CCTTCCTCATCCCCACGTCCA 3′	
PXR-F	5′CCCTCAAGAGCTACATCGACCA 3′	108bp
PXR-R	5′TGTTCTCCATCTTCAGCGTCT 3′	
CYP1A1-F	5′ATGCTCGTTTCAGTGCCTTCGT 3′	199bp
CYP1A1-R	5′GTGTCAAAGCCTGCCCCAA 3′	
CYP1A5-F	5′AACCCAGAGCGTTTCCTCAA 3′	120bp
CYP1A5-R	5′CTCCCACTTGCCTATGTTTTCC 3′	
CYP2C18-F	5′AACCTCCATACGAAGCTGCAA 3′	243bp
CYP2C18-R	5′TGTGCCTTTGAAGACTTTCTCA 3′	
CYP2D6-F	5′GAACCCTGCTTACATCCGAGA 3′	175bp
CYP2D6-R	5′CATGAACAGGAACGCCCAT 3′	
CYP3A9-F	5′GAGATGAGACTGTGAATGG 3′	199bp
CYP3A9-R	5′TTTTTTTAATGGTGATAGAAC 3′	
GRP78-F	5′GAATCGGCTAACACCAGAGGA 3′	118bp
GRP78-R	5′CGCATAGCTCTCCAGCTCATT 3′	
GRP94-F	5′CAAAGACATGCTGAGGCGAGT 3′	186bp
GRP94-R	5′TCCACCTTTGCATCCAGGTCA 3′	
ATF4-F	5′GAATCGGCTAACACCAGAGGA 3′	118bp
ATF4-R	5′CGCATAGCTCTCCAGCTCATT 3′	
ATF6-F	5′CGTCGTCTGAACCACTTACTGA 3′	101bp
ATF6-R	5′CCTTCTTTCCTAACAGCCACAC 3′	
eIF2α-F	5′CAGGGGCACCCAACCTACAA 3′	121bp
eIF2α-R	5′CGGGCACAAATACTTTCATCATCT 3′	
IRE1-F	5′CTACAGGTCGCTCCTCACATC 3′	101bp
IRE1-R	5′ATCAGTCCTTCTGCTCCCATCT 3′	
PERK-F	5′TCATCCAGCCTCAGTAAACC 3′	164bp
PERK-R	5′ACAACATCCTCGCCCAGT 3′	
CHOP-F	5′GCTGGATGAGACACTGAATGCAGAG 3′	149bp
CHOP-R	5′CACGCTTCCGCTTTGTCCTCTG 3′	
DRP1-F	5′TCGTGCTCCTCCTGGTGTTCC 3′	161bp
DRP1-R	5′TTCTGTGCGTTGCCACCGATG 3′	
MFF-F	5′GGCTCCTCAGAATGCTGACCTTG 3′	91bp
MFF-R	5′CACTACAATCCGCTCTGGAACCTG 3′	
OPA1-F	5′TGTATGTGATGAGATAGCCTGG 3′	115bp
OPA1-R	5′CCAGCTCCACTGTACAAGACA 3′	
MFN1-F	5′CCTGCTGCAACTCCAGAGAACAC 3′	115bp
MFN1-R	5′TCACTCCGCCAACAACGATGATG 3′	
MFN2-F	5′AGCTGGCTGCGTACATCAATGAG 3′	150bp

MFN2-R	5′GCCTTGCCAACACTTCACTAATGC 3′	
β-actin-F	5′CCAGCCATGTATGTAGCCATCCAG 3′	93bp
β-actin-R	5′GGTAACACCATCACCAGAGTCCAT C 3′	

Table S2. The antigens that were used in the current research.

Antibody name	Dilution ratio	kDa	Resource
GRP78	1:1000	78	Proteintech Biotechnology, China
ATF6	1:1000	100	Proteintech Biotechnology, China
CHOP	1:1000	30	Proteintech Biotechnology, China
DRP1	1:500	80	Abclonal Technology, China
OPA1	1:1000	100	Abclonal Technology, China
MFN1	1:1000	84	Abclonal Technology, China
MFN2	1:1000	86	Abclonal Technology, China
β-actin	1:1000	42	Abclonal Technology, China
